# Crosstalk between NDR kinase pathways coordinates cell cycle dependent actin rearrangements

**DOI:** 10.1186/1747-1028-6-19

**Published:** 2011-11-11

**Authors:** Sneha Gupta, Dannel McCollum

**Affiliations:** 1 Department of Microbiology and Physiological Systems, and Program in Cell Dynamics, University of Massachusetts Medical School, Worcester, Massachusetts 01605, USA

**Keywords:** SIN, MOR, polarity, cytokinesis, actin cytoskeleton

## Abstract

Regulation of cytoskeletal remodeling is essential for cell cycle transitions. In fission yeast two NDR kinase signaling cascades, MOR and SIN, regulate the actin cytoskeleton to promote polarized growth during interphase and cytokinesis respectively. Our understanding of how these signaling pathways are coordinated to assist transition between the two cell-cycle stages is limited. Here, we review work from our laboratory, which reveals that cross talk between the SIN and MOR pathways is required for inhibition of interphase polarity programs during cytokinesis. Given the conservation of NDR kinase signaling pathways, our results may define general mechanisms by which these pathways are coordinated in higher organisms.

## Introduction

**Figure 1:**
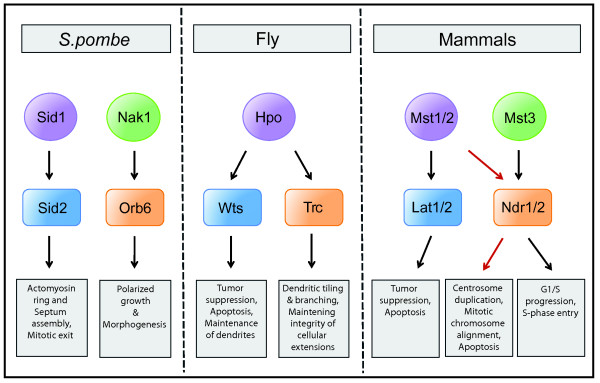
**Conservation of NDR kinase pathways across species**. Members of the Ste-20 like kinase family (Sid1, Nak1, Hpo, Mst1/2, Mst3) and the Nuclear Dbf2-related kinase family (Sid2, Orb6, Wts, Trc, Lats1/2, Ndr1/2) constitute the core of the NDR kinase signaling pathways in *S. pombe*, *D. melanogaster *(Fly), and mammals. This figure illustrates the cellular functions performed by these pathways in their respective organisms.

*Schizosaccharomyces pombe *are rod-shaped cells that grow by elongation at cell ends and divide by medial fission. These cells form an ideal system for the study of biochemical signaling pathways that underlie cell polarity and morphogenesis. The NDR kinase signaling cascades that control various aspects of growth and division in fission yeast are conserved in higher eukaryotes where they retain similar functions but have also acquired new ones (Figure 1) [1,2].

The Morphogenesis Orb6 Network (MOR), one of the two conserved NDR kinase pathways in fission yeast, is active throughout interphase and controls cell morphology and tip growth through accretion of actin to sites of cell growth [3,4]. Actin is required at the cell tips for formation of F-actin patch structures as well as actin cables. While actin patches are well established as sites of active cell growth, actin cables serve as tracks on which a variety of cargo for cell growth and polarity are delivered to the cell ends [5-7]. The MOR has been implicated in F-actin patch assembly [8]. Moreover, it also regulates localization of the F-actin cable polymerization factor, For3 at the cell tips via spatial control of the Cdc42 GTPase, a central regulator of cell polarity [9]. The MOR signaling pathway includes Orb6 (an NDR kinase) [3]; its binding partner Mob2 [10] and its upstream activator, Nak1 (a STE-20 like kinase) [11,12]. Pmo25 has been identified as a binding partner of Nak1 and is essential for the activities of both kinases in the pathway [4]. Mor2, which is a homolog of the *Drosophila *Furry protein, is thought to act as a scaffold that promotes the activation of Orb6 by Nak1 [13]. Mutants in any of the MOR components fail to grow in a polarized manner resulting in round morphology of the cells.

The Septation Initiation Network (SIN) constitutes the other NDR kinase pathway in fission yeast and is activated during late mitosis where it plays an essential role in cytokinesis. The SIN signaling cascade is regulated by an upstream GTPase, Spg1 that controls the protein kinases Cdc7, Sid1 (a STE 20-like kinase) and Sid2 (an NDR kinase) [14]. Most SIN components localize exclusively to the spindle pole body [14]. An exception to this is the Sid2 kinase, which, upon activation by Sid1, translocates from the SPB to the medial ring where it promotes assembly and constriction of the actomyosin ring as well as formation of the division septum [15]. SIN mutants are unable to maintain a stable actomyosin ring and display cytokinetic failure resulting in long multinucleate cells.

It is evident that performing the tasks of cell growth and division requires significant restructuring of the actin cytoskeleton. To promote polarized growth during interphase, actin is confined to the cell ends where it is required for cell wall deposition. As cells enter mitosis, actin relocalizes to the cell middle to form the contractile ring that marks the site of septum formation [5]. Coordination of actin localization as cells transition between interphase and mitosis is presumably important to keep competing actin polarity programs from interfering with each other. Recent work from our laboratory reveals a previously unknown link between the two NDR kinase signaling pathways in *S. pombe *(MOR and SIN) that is critical for proper regulation of actin polarity during cell cycle transitions [16].

## Discussion

### Activation of SIN during interphase disrupts MOR activity and function

During interphase growth, SIN mediated septum formation is inhibited by keeping the Spg1 GTPase in an inactive GDP-bound state. A previous study showed that constitutive SIN activation blocked cell elongation and resulted in uncontrolled septation [17]. In order to test the effect of SIN activity on polarized interphase growth, the SIN was ectopically activated in an asynchronous cells using a temperature-sensitive (ts) mutant of Cdc16, a GAP for Spg1 [18]. To ensure that ectopic septation was not responsible for the previously observed inhibition of cell elongation, the *cdc16-116 *ts mutant was combined with either a *cdc15-140 *(a PCH family protein) or a *cdc3-124 *(Profilin) ts mutant, both of which disrupt actomyosin ring and septum formation and therefore allow activation of the SIN without causing formation of ectopic septa [19,20]. Both genotypes resulted in cells that showed an arrest in cell elongation coupled with an increase in cell diameter. Actin distribution reflected this phenotype since the actin cytoskeleton was dispersed throughout the cell instead of having a polarized configuration [16]. MOR mutants show a similar actin distribution and block in cell elongation raising the possibility that the SIN disrupts the interphase actin cytoskeleton by inhibiting the MOR.

In addition to a displaying a disorganized actin cytoskeleton and an inhibition of cell elongation, MOR mutants also arrest in G2 phase. This G2 arrest was found to be dependent on Wee1 [13], a Cdc2 inhibitory kinase that prevents G2/M progression. Interestingly, ectopic activation of the SIN also resulted in a Wee1 mediated G2 arrest. While this block in mitotic progression is rescued by the *wee1-50 *mutation, these cells continue to exhibit both depolarized actin and a block in cell elongation, similar to observations in double mutants between the MOR and *wee1-50 *[13,16]. Therefore, although both SIN and MOR inhibition block cell elongation and nuclear division, the cell elongation block is not an indirect consequence of the block in nuclear division.

Our observations showed that ectopic activation of the SIN mimics the absence of MOR activity. Moreover, previous experiments monitoring the activity of the MOR pathway kinase, Orb6 through the cell cycle revealed that Orb6 activity is reduced during mitosis [4]. In order to test whether SIN inhibits MOR activity, Orb6 kinase activity was measured in the presence or absence of ectopic SIN activation. Cells with an activated SIN pathway had significantly decreased levels of Orb6 activity [16]. Together, these results suggested that the SIN prevents polarized interphase growth by reducing the activity of the MOR pathway kinase, Orb6.

### SIN prevents polarized growth through inhibition of Nak1 mediated Orb6 activation

To examine the mechanism of SIN mediated MOR inhibition further, the effect of SIN signaling on the activity of the Nak1 kinase, an upstream activator of Orb6, was measured. It has been previously shown that Nak1 activity is unchanged throughout the cell cycle [11]. Consistent with this result, we observed that Nak1 kinase activity was not affected by the SIN [16]. Since Nak1 is suggested to physically interact with and activate Orb6, it was hypothesized that SIN might inhibit association of Nak1 with Orb6. A fusion of the two protein kinases was created to test this possibility. Interestingly, expression of the Nak1-Orb6 fusion in a SIN activated background was able to bypass SIN mediated inhibition of actin polarization and cell elongation. In addition to bypassing the inhibition of cell elongation, cells expressing the Nak1-Orb6 fusion protein were also able to partially override the block in nuclear division [16]. These results indicated that polarized cell growth as well as nuclear division is prevented during cytokinesis, at least in part, through SIN mediated inhibition of the MOR pathway.

The Nak1-Orb6 fusion also provided an insight into the function of the MOR pathway protein Mor2. As observed in other organisms, Mor2 is required for Orb6 but not Nak1 kinase activity, supporting the notion that it functions as a scaffold to promote Nak1 mediated activation of Orb6 [4,21,22]. Consistent with this notion, the Nak1-Orb6 fusion was also able to rescue a mutation in Mor2 [16]. Although these studies showed that the SIN inhibits the MOR by blocking activation of Orb6 by Nak1, the exact molecular mechanisms involved in this process remain to be uncovered.

### Inhibition of the MOR pathway during cytokinesis is an important SIN function

SIN activity is essential to promote assembly and maintenance of a stable actomyosin ring in the cell middle as well as to prevent polarization of F-actin at the cell tips [14,23]. Our observations suggest that the SIN inhibits localization of actin to cell tips by directly inhibiting MOR activity [16]. Since bypass of SIN inhibition of the MOR restores actin localization to the tips, it seemed plausible that inhibition of the MOR during cytokinesis may be necessary to prevent competition for cytoskeletal components such as actin that are shared by the two pathways. If this were true, inability to execute this inhibition would severely affect the process of cytokinesis. Although expression of the Nak1-Orb6 fusion in wild-type cells had only mild effects, these cells were sensitive to treatment with low doses of the actin depolymerizing drug, LatB which impairs cytokinesis. LatB treatment causes a cell division delay in wild-type cells resulting in an increase in the number of binucleate cells [24]. In the presence of the fusion however, these cells failed cytokinesis entirely resulting in accumulation of multinucleate cells. In addition, expression of the Nak1-Orb6 fusion in a weakened SIN background resulted in a lethal phenotype [16]. Together, these experiments demonstrated that inhibition of MOR is essential for cytokinesis when SIN signaling or the cytokinetic apparatus is perturbed. The inability of the fusion to cause a substantial effect in wild-type cells could indicate that failure to inhibit the MOR makes cytokinesis less robust and more sensitive to perturbation. Alternatively, the Nak1-Orb6 fusion may only partially bypass SIN inhibition in wild-type cells suggesting that the inability of SIN to inhibit MOR could have larger implications on SIN function and its complete impact remains to be tested.

To further understand the interplay between SIN and MOR signaling, the effect of reducing MOR activity on SIN function was tested. This analysis showed that reducing MOR pathway activity partially rescued viability as well as defects in actomyosin ring constriction and septum formation in SIN mutants. Furthermore, weak activation of the SIN was able to trigger ectopic cytokinesis in MOR mutants but not wild-type cells [16]. Together, these observations showed that a major function of the SIN is to inhibit the MOR. Also, the MOR did not appear to antagonize the ability of the SIN to promote cytokinesis by directly inhibiting the SIN since the Nak1-Orb6 fusion did not impair SIN signaling [16]. Thus, SIN inhibition of MOR during cytokinesis is important for performing downstream functions of SIN but does not appear to be required for maintenance of SIN activity.

### Crosstalk observed between conserved NDR kinase pathways in other eukaryotic systems

**Figure 2:**
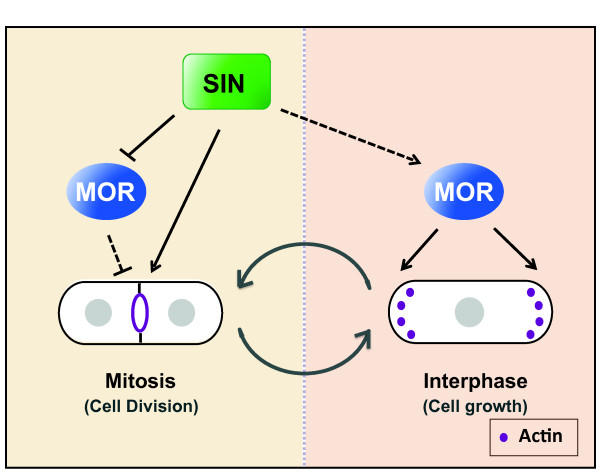
**A dual role for the SIN in MOR pathway regulation**. The SIN mediated regulation of MOR during mitosis appears to have two distinct cell cycle dependent effects on MOR function. While the SIN inhibits MOR activity during mitosis to keep MOR from interfering with cytokinesis, it may also play a role in enhancing MOR activity during the following interphase in order promote polarized growth.

Our study in *S. pombe *confirms that opposing effects of the SIN and MOR networks on regulation of cell growth and division necessitates the presence of an antagonistic interaction between the two pathways (Figure 2). From work in other model systems it is now evident that homologous NDR kinase pathways have contrasting functions in various cellular processes. For instance, recent observations in *Drosophila melanogaster *indicates that the NDR kinases, Trc (Orb6 homolog) and Wts (Sid2 homolog) have opposing roles in regulation of cell shape and timing of hair morphogenesis in wing cells [25]. Furthermore, various studies in mammalian systems have shown that their SIN and MOR counterparts, namely, the MST1/2-LATS1/2 and MST3-NDR1/2 signaling pathways have contradictory effects on cell proliferation [1,26,27]. These observations suggest the possibility that an antagonistic crosstalk similar to the one observed in fission yeast may exist between homologous NDR kinase pathways in higher organisms.

Alternatively, there are several examples where the two NDR pathways work in concert to promote common cellular functions. In the budding yeast *S. cerevisiae*, the MEN (Mitosis Exit Network) and RAM (Regulation of Ace2p and morphogenesis) signaling networks correspond to the *S. pombe *pathways SIN and MOR respectively [28,29]. It has been reported that MEN and RAM function together to regulate the function of the Ace2 transcription factor in daughter cell separation [30]. In *Drosophila*, the two NDR kinases Trc and Wts share the same upstream regulator Hippo (Hpo), which may help coordinate their roles in the establishment and maintenance of dendritic tiling in neuronal cells [31,32]. While the MST1/2-LATS1/2 pathway in mammals plays a role in tumor suppression and growth inhibition, several recent reports now implicate MST1/2 in the additional regulation of NDR1/2 kinases to control various cellular processes like centrosome duplication, mitotic chromosome alignment, and apoptotic signaling [33-35]. In contrast, Mst3 kinase appears to be important for the growth promoting functions of Ndr1/2 [36]. Therefore, in animal systems, the regulation of NDR kinases functioning in separate pathways by a common upstream kinase of the STE20-like kinase family appears to be a conserved characteristic (Figure 1). Intriguingly, a similar regulation has been proposed by a study in *S. pombe*, which indicates that the SIN kinase Sid1 not only regulates cytokinesis through activation of Sid2 but also functions in enhancement of MOR activity during the subsequent interphase [4] (Figure 2). It is possible that SIN imparts both activating and inhibitory modifications on its MOR targets. While the inhibitory regulation dominates during mitosis, its removal in the subsequent interphase could result in MOR activation. Taken together with our findings, it reveals a dual role for the SIN in regulation of the MOR pathway that enables it to modulate MOR activity in accordance with the cell cycle stage (Figure 2).

## Conclusion

In fission yeast, NDR kinases constitute central effectors of the MOR and SIN pathways. Our studies have uncovered a mechanism by which these pathways communicate in order to achieve sequential reorganization of the actin cytoskeleton during the cell cycle. Both NDR signaling pathways in *S. pombe *are conserved in higher eukaryotes, where several studies have provided a flurry of information emphasizing their biological relevance. For instance, in drosophila and mammalian systems, NDR kinase homologs Wts/Lat1/2 and Trc/NDR1/2 act as tumor suppressors and proto-oncogenes in addition to having conserved cell cycle functions in morphogenesis and mitotic exit. Their regulation of targets like p21 and c-Myc makes them highly appealing candidates for cancer therapy [1]. However, the presence of multiple isoforms of core components of the pathway like the Ste-20 like kinases, scaffold proteins, and MOB activators adds to the diversity of their biological functions; making it difficult to dissect these complex signaling networks in mammals. Therefore, examination of these pathways in relatively simple model systems like *S. pombe *can provide highly useful cues enabling us to pursue a more clear understanding of their functions in humans.

## List of Abbreviations

SIN: Septation Initiation Network; MOR: Morphogenesis Orb6 Network, LatB: Latrunculin B; NDR: Nuclear Dbf2 Related; MEN: Mitosis Ext Network; RAM: Regulation of Ace2 and morphogenesis; GAP: GTPase Activating Protein; PCH: Pombe Cdc15 Homology; MOB: Mps One Binder; MST: Mammalian sterile 20-like

## Competing interests

The authors declare that they have no competing interests.

## Authors' contributions

Both authors drafted, read and approved the manuscript.
